# Ovary and uterus related adverse events associated with statin use: an analysis of the FDA Adverse Event Reporting System

**DOI:** 10.1038/s41598-020-68906-2

**Published:** 2020-07-20

**Authors:** Xue-feng Jiao, Hai-long Li, Xue-yan Jiao, Yuan-chao Guo, Chuan Zhang, Chun-song Yang, Li-nan Zeng, Zhen-yan Bo, Zhe Chen, Hai-bo Song, Ling-li Zhang

**Affiliations:** 1 Department of Pharmacy, West China Second University Hospital, Sichuan University, Chengdu, 610041 Sichuan China; 2 Evidence-Based Pharmacy Center, West China Second University Hospital, Sichuan University, Chengdu, 610041 Sichuan China; 3 Key Laboratory of Birth Defects and Related Diseases of Women and Children (Sichuan University), Ministry of Education, Chengdu, 610041 Sichuan China; 4 West China School of Medicine, Sichuan University, Chengdu, 610041 Sichuan China; 5 Xinxiang Medical University, Xinxiang, 453000 Henan China; 6 Center for Drug Reevaluation, National Medical Products Administration, Beijing, 100000 China

**Keywords:** Endocrine reproductive disorders, Gonadal disorders, Reproductive disorders, Risk factors, Adverse effects

## Abstract

Experimental studies have demonstrated statin-induced toxicity for ovary and uterus. However, the safety of statins on the functions of ovary and uterus in real-world clinical settings remains unknown. The aim of this study was to identify ovary and uterus related adverse events (AEs) associated with statin use by analyzing data from FDA Adverse Event Reporting System (FAERS). We used OpenVigil 2.1 to query FAERS database. Ovary and uterus related AEs were defined by 383 Preferred Terms, which could be classified into ten aspects. Disproportionality analysis was performed to assess the association between AEs and statin use. Our results suggest that statin use may be associated with a series of ovary and uterus related AEs. These AEs are involved in ovarian cysts and neoplasms, uterine neoplasms, cervix neoplasms, uterine disorders (excl neoplasms), cervix disorders (excl neoplasms), endocrine disorders of gonadal function, menstrual cycle and uterine bleeding disorders, menopause related conditions, and sexual function disorders. Moreover, there are variabilities in the types and signal strengths of ovary and uterus related AEs across individual statins. According to our findings, the potential ovary and uterus related AEs of statins should attract enough attention and be closely monitored in future clinical practice.

## Introduction

Statins, inhibitors of 3-hydroxy-3-methyl-glutaryl coenzyme A (HMG-CoA) reductase, reduce the biosynthesis of cholesterol by blocking the mevalonate pathway. As such they are prescribed for patients with high cholesterol levels who have or are at risk for coronary atherosclerotic heart disease. Statins are among the most widely prescribed medicines in the United States and other countries^1,2^. For example, in America alone, the number of statin users is estimated at more than 38 million^3^.

Besides prevention of cardiovascular disease, statins have therapeutic effects in several ovary and uterus related diseases, such as polycystic ovary syndrome^4^, ovarian cancer^5^, endometrial cancer^6^, uterine fibroids^7^, and endometriosis^8^. But meanwhile, concerns have been raised about potential toxicity for ovary and uterus associated with statin use. Currently, experimental studies of human cell lines and animal models have demonstrated the toxic effects of statins on the functions of ovary and uterus. These toxic effects include the inhibition of ovarian steroidogenesis, the anti-proliferative and pro-apoptotic effects on ovarian cells and endometrial cells, the morphological and histological changes of ovary, and the reduction in fertility^8,9^.

However, published data about the safety of statins on the functions of ovary and uterus in clinical studies are scarce, and full of controversy. A few clinical studies have investigated the effects of statins on gonadal steroid hormones, but generated conflicting results: some indicated that statins could reduce serum androgen levels^4^, while others found no effects on gonadal steroid hormones^10,11^. However, these clinical studies were all limited by either small sample size or short length of statin exposure, and none of them had sufficient power to ascertain the effects of statins on gonadal steroid hormones. Only one randomized controlled trial (RCT) assessed the safety of statins on ovarian function using the change in luteal phase duration as the primary outcome^12^. However, this study was also influenced by several biases linked to small sample size (86 women), short length of statin exposure (only 4 months), and selection of primary outcome. Due to the limited quantity and quality of current clinical studies, the safety of statins on the functions of ovary and uterus remains unknown, and there may exist some unknown ovary and uterus related adverse reactions occurring in real-world clinical settings. The FDA Adverse Event Reporting System (FAERS) is the largest spontaneous reporting database in the world which contains more than sixteen million adverse event (AE) reports submitted to the FDA, and could reflect complete AE profiles in real-world clinical settings^13^. Moreover, data mining algorithms have already been developed for signal detection in this database, that is, a “positive signal” means a statistical association between an AE and a drug^14^. With these advantages, the FAERS database is now widely used to detect potential drug-associated AEs^15,16^. Thus, in this study, we aimed to identify ovary and uterus related AEs associated with statin use by analyzing data from FAERS.

## Results

### Overview of female AE reports submitted for statins

The overview of female AE reports submitted for statins in FAERS is shown in Table 1. The numbers of AE reports for statins as a class, atorvastatin, simvastatin, rosuvastatin, pravastatin, lovastatin, fluvastatin and pitavastatin were 151,924, 64,177, 44,586, 28,991, 10,810, 4,965, 1,591 and 916 respectively. When stratified by age group, the majority of AE reports were distributed in the 40–59 years and ≥ 60 years age groups for both statins as a class and individual statins. After disproportionality analyses, the numbers of ovary and uterus related PTs with positive signals for statins as a class, atorvastatin, simvastatin, rosuvastatin, pravastatin, lovastatin, fluvastatin and pitavastatin were 15, 12, 19, 3, 6, 5, 5 and 0, respectively.

**Table 1:** Overview of female adverse event reports submitted for statins in FAERS.

Counting numbers by age groups (years)	Statins as a class	Atorvastatin	Simvastatin	Rosuvastatin	Pravastatin	Lovastatin	Fluvastatin	Pitavastatin
**Number of AE reports (n, %)**								
Overall	151,924	64,177	44,586	28,991	10,810	4,965	1,591	916
0–18	279 (0.18%)	102 (0.16%)	74 (0.17%)	39 (0.13%)	30 (0.28%)	25 (0.50%)	5 (0.31%)	2 (0.22%)
19–39	2,663 (1.75%)	1,034 (1.61%)	915 (2.05%)	416 (1.43%)	197 (1.82%)	87 (1.75%)	48 (3.02%)	10 (1.09%)
40–59	30,198 (19.88%)	12,287 (19.15%)	9,150 (20.52%)	6,049 (20.87%)	2096 (19.39%)	1,082 (21.79%)	372 (23.38%)	167 (18.23%)
≥ 60	73,777 (48.56%)	30,003 (46.75%)	22,203 (49.80%)	14,032 (48.40%)	5,618 (51.97%)	2,344 (47.21%)	800 (50.28%)	528 (57.64%)
**Number of ovary and uterus related PTs with positive signals (n)**								
Overall	2	6	9	2	3	2	5	0
0–18	1	0	0	0	0	0	0	0
19–39	3	1	2	1	1	0	0	0
40–59	6	5	9	2	2	4	2	0
≥ 60	6	5	8	0	1	1	3	0
Total number	15	12	19	3	6	5	5	0

*FAERS* FDA Adverse Event Reporting System, *AE* adverse event, *PTs* preferred terms.

### Positive signals for statins as a class

For all females, statistically significant signals emerged in two ovary and uterus related PTs: ovarian adenoma and androgenetic alopecia. For the 0–18 years age group, statistically significant signals emerged in one more PT: precocious puberty. For the 19–39 years age group, statistically significant signals emerged in three more PTs: endometrial hyperplasia, cervical incompetence, and polycystic ovaries. For the 40–59 years age group, statistically significant signals emerged in five more PTs: adenocarcinoma of the cervix, cervix carcinoma stage III, menopausal symptoms, disturbance in sexual arousal, and orgasm abnormal. For the ≥ 60 years age group, statistically significant signals emerged in four more PTs: cervix disorder, dysfunctional uterine bleeding, dyspareunia, and sexual dysfunction. In total, the above 15 PTs with positive signals were involved in ovarian cysts and neoplasms, cervix neoplasms, uterine disorders (excl neoplasms), cervix disorders (excl neoplasms), endocrine disorders of gonadal function, menstrual cycle and uterine bleeding disorders, menopause related conditions, and sexual function disorders (Table 2).

**Table 2:** Ovary and uterus related adverse events with positive signals for statins as a class.

Classification	PTs	Proportional rate ratio (PRR) by age groups (years)														
		Overall			0–18			19–39			40–59			≥ 60		
		N	PRR	χ^2^	N	PRR	χ^2^	N	PRR	χ^2^	N	PRR	χ^2^	N	PRR	χ^2^
Ovarian cysts and neoplasms	Ovarian adenoma	30	9.83	165.77	–	–	–	–	–	–	–	–	–	29	27.35	219.18
Cervix neoplasms	Adenocarcinoma of the cervix	–	–	–	–	–	–	–	–	–	3	9.66	11.03	–	–	–
	Cervix carcinoma stage III	–	–	–	–	–	–	–	–	–	3	5.52	5.69	–	–	–
Uterine disorders (excl neoplasms)	Endometrial hyperplasia	–	–	–	–	–	–	3	8.40	12.22	–	–	–	–	–	–
Cervix disorders (excl neoplasms)	Cervix disorder	–	–	–	–	–	–	–	–	–	–	–	–	4	7.01	9.30
	Cervical incompetence	–	–	–	–	–	–	3	4.50	4.90	–	–	–	–	–	–
Endocrine disorders of gonadal function	Androgenetic alopecia	21	13.25	149.00	–	–	–	–	–	–	12	17.18	99.86	7	14.31	33.62
	Polycystic ovaries	–	–	–	–	–	–	5	4.10	8.60	–	–	–	–	–	–
	Precocious puberty	–	–	–	3	11.05	17.82	–	–	–	–	–	–	–	–	–
Menstrual cycle and uterine bleeding disorders	Dysfunctional uterine bleeding	–	–	–	–	–	–	–	–	–	–	–	–	4	4.91	6.12
Menopause related conditions	Menopausal symptoms	–	–	–	–	–	–	–	–	–	35	2.73	32.97	–	–	–
Sexual function disorders	Dyspareunia	–	–	–	–	–	–	–	–	–	–	–	–	7	4.29	10.59
	Sexual dysfunction	–	–	–	–	–	–	–	–	–	–	–	–	9	2.40	4.94
	Disturbance in sexual arousal	–	–	–	–	–	–	–	–	–	6	3.44	7.05	–	–	–
	Orgasm abnormal	–	–	–	–	–	–	–	–	–	4	4.91	7.32	–	–	–

*PTs* preferred terms, *PRR* proportional reporting ratio, *χ*^*2*^ chi-square, – not a positive signal.

### Positive signals for atorvastatin

For all females, statistically significant signals emerged in six ovary and uterus related PTs: ovarian cancer stage I, ovarian germ cell teratoma benign, endometrial disorder, adenomyosis, androgenetic alopecia, and blood testosterone increased. For the 19–39 years age group, statistically significant signals emerged in one more PT: endometriosis. For the 40–59 years age group, statistically significant signals emerged in three more PTs: menopausal symptoms, dyspareunia, and anorgasmia. For the ≥ 60 years age group, statistically significant signals emerged in two more PTs: uterine haemorrhage and sexual dysfunction. In total, the above 12 PTs with positive signals were involved in ovarian cysts and neoplasms, uterine disorders (excl neoplasms), endocrine disorders of gonadal function, menstrual cycle and uterine bleeding disorders, menopause related conditions, and sexual function disorders (Table 3).

**Table 3:** Ovary and uterus related adverse events with positive signals for atorvastatin.

Classification	PTs	Overall			0–18			19–39			40–59			≥ 60		
		N	PRR	χ^2^	N	PRR	χ^2^	N	PRR	χ^2^	N	PRR	χ^2^	N	PRR	χ^2^
Ovarian cysts and neoplasms	Ovarian cancer stage I	4	7.25	14.01	–	–	–	–	–	–	–	–	–	–	–	–
	Ovarian germ cell teratoma benign	4	5.24	9.00	–	–	–	–	–	–	–	–	–	–	–	–
Uterine disorders (excl neoplasms)	Endometrial disorder	8	2.55	5.82	–	–	–	–	–	–	–	–	–	5	3.46	5.75
	Adenomyosis	9	2.33	5.34	–	–	–	–	–	–	–	–	–	–	–	–
	Endometriosis	–	–	–	–	–	–	5	4.24	9.26	–	–	–	5	4.32	8.40
Endocrine disorders of gonadal function	Androgenetic alopecia	14	18.35	163.63	–	–	–	–	–	–	12	42.36	266.11	–	–	–
	Blood testosterone increased	9	3.36	11.97	–	–	–	–	–	–	5	11.77	32.85	–	–	–
Menstrual cycle and uterine bleeding disorders	Uterine haemorrhage	–	–	–	–	–	–	–	–	–	–	–	–	9	2.86	8.28
Menopause related conditions	Menopausal symptoms	–	–	–	–	–	–	–	–	–	18	3.32	25.48	–	–	–
Sexual function disorders	Dyspareunia	–	–	–	–	–	–	–	–	–	8	2.82	7.34	6	8.89	26.66
	Sexual dysfunction	–	–	–	–	–	–	–	–	–	–	–	–	8	5.30	20.20
	Anorgasmia	–	–	–	–	–	–	–	–	–	8	3.20	9.48	–	–	–

*PTs* preferred terms,* PRR* proportional reporting ratio,* χ*^2^: chi-square,* —* not a positive signal.

### Positive signals for simvastatin

For all females, statistically significant signals emerged in nine ovary and uterus related PTs: ovarian adenoma, uterine polyp, adenocarcinoma of the cervix, cervical polyp, uterine prolapse, androgenetic alopecia, menopausal symptoms, postmenopausal haemorrhage, and disturbance in sexual arousal. For the 19–39 years age group, statistically significant signals emerged in two more PTs: cervix carcinoma and polycystic ovaries. For the 40–59 years age group, statistically significant signals emerged in four more PTs: endometriosis, cervical dysplasia, libido increased, and sexual dysfunction. For the ≥ 60 years age group, statistically significant signals emerged in four more PTs: uterine leiomyoma, endometrial hypertrophy, uterine mass, and menstruation irregular. In total, the above 19 PTs with positive signals were involved in ovarian cysts and neoplasms, uterine neoplasms, cervix neoplasms, uterine disorders (excl neoplasms), cervix disorders (excl neoplasms), endocrine disorders of gonadal function, menstrual cycle and uterine bleeding disorders, menopause related conditions, and sexual function disorders (Table 4).

**Table 4:** Ovary and uterus related adverse events with positive signals for simvastatin.

Classification	PTs	Proportional rate ratio (PRR) by age groups (years)														
		Overall			0–18			19–39			40–59			≥ 60		
		N	PRR	χ^2^	N	PRR	χ^2^	N	PRR	χ^2^	N	PRR	χ^2^	N	PRR	χ^2^
Ovarian cysts and neoplasms	Ovarian adenoma	29	33.13	635.28	–	–	–	–	–	–	–	–	–	29	94.60	801.96
Uterine neoplasms	Uterine polyp	18	2.12	9.28	–	–	–	–	–	–	–	–	–	11	3.62	16.80
	Uterine leiomyoma	–	–	–	–	–	–	–	–	–	–	–	–	12	2.19	6.30
Cervix neoplasms	Cervix carcinoma	–	–	–	–	–	–	3	6,21	8.30	–	–	–	–	–	–
	Adenocarcinoma of the cervix	3	6.52	8.38	–	–	–	–	–	–	3	32.13	44.89	–	–	–
	Cervical polyp	6	3.30	7.17	–	–	–	–	–	–	–	–	–	–	–	–
Uterine disorders (excl neoplasms)	Uterine prolapse	11	2.22	6.03	–	–	–	–	–	–	–	–	–	–	–	–
	Endometrial hypertrophy	–	–	–	–	–	–	–	–	–	–	–	–	5	3.66	6.56
	Endometriosis	–	–	–	–	–	–	–	–	–	9	2.37	5.67	–	–	–
	Uterine mass	–	–	–	–	–	–	–	–	–	–	–	–	3	6.70	8.02
Cervix disorders (excl neoplasms)	Cervical dysplasia	–	–	–	–	–	–	–	–	–	7	2.84	6.39	4	4.71	7.39
Endocrine disorders of gonadal function	Androgenetic alopecia	18	36.45	415.40	–	–	–	–	–	–	12	57.11	353.61	5	26.51	60.31
	Polycystic ovaries	–	–	–	–	–	–	4	9.40	21.79	3	7.14	9.45	–	–	–
Menstrual cycle and uterine bleeding disorders	Menstruation irregular	–	–	–	–	–	–	–	–	–	–	–	–	3	12.72	16.55
Menopause related conditions	Menopausal symptoms	21	2.37	14.91	–	–	–	–	–	–	14	3.44	20.95	–	–	–
	Postmenopausal haemorrhage	23	2.50	18.68	–	–	–	–	–	–	–	–	–	–	–	–
Sexual function disorders	Libido increased	–	–	–	–	–	–	–	–	–	4	3.46	4.55	–	–	–
	Sexual dysfunction	–	–	–	–	–	–	–	–	–	7	2.43	4.40	–	–	–
	Disturbance in sexual arousal	7	4.55	15,21	–	–	–	–	–	–	5	9.31	26.31	–	–	–

*PTs* preferred terms, *PRR* proportional reporting ratio, *χ*^*2*^ chi-square, – not a positive signal.

### Positive signals for rosuvastatin

For all females, statistically significant signals emerged in two ovary and uterus related PTs: cervix carcinoma stage III and endometrial hypertrophy. For the 19–39 years age group, statistically significant signals emerged in one more PT: uterine leiomyoma. In total, the above three PTs with positive signals were involved in uterine neoplasms, cervix neoplasms, and uterine disorders (excl neoplasms) (Table 5).

**Table 5:** Ovary and uterus related adverse events with positive signals for rosuvastatin.

Classification	PTs	Proportional rate ratio (PRR) by age groups (years)														
		Overall			0–18			19–39			40–59			≥ 60		
		N	PRR	χ^2^	N	PRR	χ^2^	N	PRR	χ^2^	N	PRR	χ^2^	N	PRR	χ^2^
Uterine neoplasms	Uterine leiomyoma	–	–	–	–	–	–	3	5.01	6.00	–	–	–	–	–	–
Cervix neoplasms	Cervix carcinoma stage III	3	10.86	16.54	–	–	–	–	–	–	3	27.88	43.66	–	–	–
Uterine disorders (excl neoplasms)	Endometrial hypertrophy	6	3.06	6.23	–	–	–	–	–	–	4	4.77	8.14	–	–	–

*PTs* preferred terms, *PRR* proportional reporting ratio, *χ*^*2*^ chi-square, – not a positive signal.

### Positive signals for pravastatin

For all females, statistically significant signals emerged in three ovary and uterus related PTs: uterine prolapse, endometrial hypertrophy, and cervical incompetence. For the 40–59 years age group, statistically significant signals emerged in two more PTs: ovarian cyst and menorrhagia. For the ≥ 60 years age group, statistically significant signals emerged in one more PT: dysfunctional uterine bleeding. In total, the above six PTs with positive signals were involved in ovarian cysts and neoplasms, uterine disorders (excl neoplasms), cervix disorders (excl neoplasms), and menstrual cycle and uterine bleeding disorders (Table 6).

**Table 6:** Ovary and uterus related adverse events with positive signals for pravastatin.

Classification	PTs	Proportional rate ratio (PRR) by age groups (years)														
		Overall			0–18			19–39			40–59			≥ 60		
		N	PRR	χ^2^	N	PRR	χ^2^	N	PRR	χ^2^	N	PRR	χ^2^	N	PRR	χ^2^
Ovarian cysts and neoplasms	Ovarian cyst	–	–	–	–	–	–	–	–	–	13	4.81	35.15	–	–	–
Uterine disorders (excl neoplasms)	Uterine prolapse	4	3.30	4.28	–	–	–	–	–	–	–	–	–	–	–	–
	Endometrial hypertrophy	3	4.07	4.17	–	–	–	–	–	–	–	–	–	–	–	–
Cervix disorders (excl neoplasms)	Cervical incompetence	3	6.62	9.07	–	–	–	3	60.59	118.10	–	–	–	–	–	–
Menstrual cycle and uterine bleeding disorders	Menorrhagia	–	–	–	–	–	–	–	–	–	10	2.01	4.09	–	–	–
	Dysfunctional uterine bleeding	–	–	–	–	–	–	–	–	–	–	–	–	4	68.22	144.03

*PTs* preferred terms, *PRR* proportional reporting ratio, *χ*^*2*^ chi-square, – not a positive signal.

### Positive signals for lovastatin

For all females, statistically significant signals emerged in two ovary and uterus related PTs: endometrial disorder and postmenopausal haemorrhage. For the 40–59 years age group, statistically significant signals emerged in three more PTs: ovarian cyst, endometrial cancer, and uterine haemorrhage. In total, the above five PTs with positive signals were involved in ovarian cysts and neoplasms, uterine neoplasms, uterine disorders (excl neoplasms), menstrual cycle and uterine bleeding disorders, and menopause related conditions (Table 7).

**Table 7:** Ovary and uterus related adverse events with positive signals for lovastatin.

Classification	PTs	Proportional rate ratio (PRR) by age groups (years)														
		Overall			0–18			19–39			40–59			≥ 60		
		N	PRR	χ^2^	N	PRR	χ^2^	N	PRR	χ^2^	N	PRR	χ^2^	N	PRR	χ^2^
Ovarian cysts and neoplasms	Ovarian cyst	–	–	–	–	–	–	–	–	–	5	3.56	6.81	–	–	–
Uterine neoplasms	Endometrial cancer	–	–	–	–	–	–	–	–	–	3	7.76	11.43	–	–	–
Uterine disorders (excl neoplasms)	Endometrial disorder	5	20.62	72.86	–	–	–	–	–	–	–	–	–	3	26.18	46.63
Menstrual cycle and uterine bleeding disorders	Uterine haemorrhage	–	–	–	–	–	–	–	–	–	3	4.51	5.03	–	–	–
Menopause related conditions	Postmenopausal haemorrhage	5	4.82	11.50	–	–	–	–	–	–	4	7.98	17.74	–	–	–

*PTs* preferred terms, *PRR* proportional reporting ratio, *χ*^*2*^ chi-square, – not a positive signal.

### Positive signals for fluvastatin

For all females, statistically significant signals emerged in five ovary and uterus related PTs: uterine polyp, uterine cancer, uterine leiomyoma, endometrial hypertrophy, and postmenopausal haemorrhage. The above five PTs with positive signals were involved in uterine neoplasms, uterine disorders (excl neoplasms), and menopause related conditions (Table 8).

**Table 8:** Ovary and uterus related adverse events with positive signals for fluvastatin.

Classification	PTs	Proportional rate ratio (PRR) by age groups (years)														
		Overall			0–18			19–39			40–59			≥ 60		
		N	PRR	χ^2^	N	PRR	χ^2^	N	PRR	χ^2^	N	PRR	χ^2^	N	PRR	χ^2^
Uterine neoplasms	Uterine polyp	3	9.80	15.66	–	–	–	–	–	–	–	–	–	3	26.36	48.95
	Uterine cancer	3	5.85	7.67	–	–	–	–	–	–	–	–	–	–	–	–
	Uterine leiomyoma	9	6.78	38.69	–	–	–	–	–	–	5	6.84	19.40	3	14.98	26.09
Uterine disorders (excl neoplasms)	Endometrial hypertrophy	3	27.74	52.32	–	–	–	–	–	–	3	58.11	113.03	–	–	–
Menopause related conditions	Postmenopausal haemorrhage	4	12.03	30.05	–	–	–	–	–	–	–	–	–	3	14.16	24.43

*PTs* preferred terms, *PRR* proportional reporting ratio, *χ*^*2*^ chi-square, – not a positive signal.

### Positive signals for pitavastatin

For both all females and each age group, there were no statistically significant signals emerged in any ovary and uterus related PT.

### Comparison of signal strength for each statin

Nine ovary and uterus related PTs with positive signals were detected in more than one statin. There was variability in signal ranking for these PTs across individual statins. Simvastatin had the strongest signal for androgenetic alopecia [PRR = 36.45 (overall); PRR = 57.11 (40–59 years)]. Pravastatin had the strongest signals for uterine prolapse [PRR = 3.30 (overall)] and ovarian cyst [PRR = 4.81 (40–59 years)]. Lovastatin had the strongest signal for endometrial disorder [PRR = 20.62 (overall); PRR = 26.18 (≥ 60 years)]. Fluvastatin had the strongest signals for uterine polyp [PRR = 9.80 (overall); PRR = 26.36 (≥ 60 years)], endometrial hypertrophy [PRR = 27.74 (overall); PRR = 58.11 (40–59 years)], postmenopausal haemorrhage [PRR = 12.03 (overall)], and uterine leiomyoma [PRR = 14,98 (≥ 60 years)]. Moreover, the signals for menopausal symptoms were comparable between atorvastatin and simvastatin (Table 9).

**Table 9:** Comparison of proportional reporting ratio (PRR) for each statin.

Age groups (years)	PTs	Atorvastatin	Simvastatin	Rosuvastatin	Pravastatin	Lovastatin	Fluvastatin
		PRR	PRR	PRR	PRR	PRR	PRR
Overall	Uterine polyp	–	2.12	–	–	–	9.80
	Endometrial disorder	2.55	–	–	–	20.62	–
	Endometrial hypertrophy	–	–	3.06	4.07	–	27.74
	Uterine prolapse	–	2.22	–	3.30	–	–
	Androgenetic alopecia	18.35	36.45	–	–	–	–
	Postmenopausal haemorrhage	–	2.50	–	–	4.82	12.03
40–59	Ovarian cyst	–	–	–	4.81	3.56	–
	Endometrial hypertrophy	–	–	4.77	–	–	58.11
	Androgenetic alopecia	42.36	57.11	–	–	–	–
	Menopausal symptoms	3.32	3.44	–	–	–	–
≥ 60	Uterine polyp	–	3.26	–	–	–	26.36
	Uterine leiomyoma	–	2.19	–	–	–	14.98
	Endometrial disorder	3.46	–	–	–	26.18	–

*PTs* preferred terms, *PRR* proportional reporting ratio, – not a positive signal.

## Discussion

To our knowledge, this is the first study to identify ovary and uterus related AEs associated with statin use by analyzing FAERS data. In our study, positive signals emerged in 15 PTs for statins as a class, 12 PTs for atorvastatin, 19 PTs for simvastatin, 3 PTs for rosuvastatin, 6 PTs for pravastatin, 5 PTs for lovastatin, and 5 PTs for fluvastatin. These PTs were involved in ovarian cysts and neoplasms, uterine neoplasms, cervix neoplasms, uterine disorders (excl neoplasms), cervix disorders (excl neoplasms), endocrine disorders of gonadal function, menstrual cycle and uterine bleeding disorders, menopause related conditions, and sexual function disorders. Moreover, there were variabilities in the types and signal strengths of ovary and uterus related PTs across individual statins.

The relationship between statin use and risk of female reproductive organ cancer is an intensely disputed topic. Several epidemiologic studies have investigated the association between statin use and the risk of ovarian and endometrial cancers, but generated conflicting results: some studies reported a reduced risk^17,18^, one study reported an increased risk^19^, and others found no correlation^20,21^. Our results indicated that statins as a class were associated with increased risk of adenocarcinoma of the cervix and cervix carcinoma stage III, atorvastatin was associated with an increased risk of ovarian cancer stage I, simvastatin was associated with increased risk of cervix carcinoma and adenocarcinoma of the cervix, rosuvastatin was associated with an increased risk of cervix carcinoma stage III, lovastatin was associated with an increased risk of endometrial cancer, and fluvastatin was associated with an increased risk of uterine cancer. Several possible mechanisms could explain our results. First, statin-induced reduction of serum cholesterol levels might be associated with increased risk of cancer^22^. Second, statins increase mitotic abnormalities, which might interfere with the development and function of centromeres, resulting in increased risk of mutations and cancer^23^. Third, statins could increase the number of regulatory T cells, which might impair the host antitumor immune response^24^.

In our study, a surprising finding was that simvastatin, rosuvastatin, and fluvastatin were associated with an increased risk of uterine leiomyoma. Contrary to our finding, a previous nested case–control study indicated that statin use was associated with a reduced risk of uterine leiomyoma^25^. In this study, the correlation was analyzed with statins as a class. However, in our study, when we examined the association between risk of uterine leiomyoma and individual statins separately, we found that simvastatin, rosuvastatin and fluvastatin had an increased risk, while other statins had no effect. Thus, the inconsistency between the results of our study and the previous one may be explained by the differential effects of individual statins on the risk of uterine leiomyoma.

Another novel finding was that statins were associated with several endometrium related AEs. For example, statins as a class were associated with an increased risk of endometrial hyperplasia. For individual statins, atorvastatin was associated with increased risk of endometrial disorder, adenomyosis and endometriosis, simvastatin was associated with increased risk of endometrial hypertrophy and endometriosis, lovastatin was associated with an increased risk of endometrial disorder, and rosuvastatin, pravastatin and fluvastatin were associated with an increased risk of endometrial hypertrophy. Several experimental studies have investigated the potential effects of statins on endometrium. These studies found that statins could inhibit the proliferation, viability and migration of endometrial cells, induce cell apoptosis and differentiation, alter cell morphology and contractility, inhibit angiogenesis, and exert anti-inflammatory effects^8,26,27^. However, these findings cannot explain some of our results clearly, and thus further studies are still needed to explore the possible mechanisms. Moreover, since 2006, encouraging results derived from both in vitro and in vivo studies seem to nominate statins as promising novel candidates for the treatment of endometriosis^28,29^. However, according to our results, the endometrium related AEs of statins should attract enough attention and call for a thorough re-examination of the efficacy and safety of statins in treating endometriosis.

Our study also found that statins as a class were associated with increased risk of androgenetic alopecia and polycystic ovaries, atorvastatin was associated with increased risk of androgenetic alopecia and blood testosterone increased, and simvastatin was associated with increased risk of androgenetic alopecia and polycystic ovaries. Interestingly, these AEs were all associated with androgen excess^30,31^. However, some previous studies indicated that statins could reduce serum androgen levels^4,32^, which seems paradoxical to interpret our results. Thus, further studies are needed to both confirm our results and explore the underlying mechanisms.

For menstrual cycle and uterine bleeding disorders, our study found that statins as a class were associated with an increased risk of dysfunctional uterine bleeding, atorvastatin and lovastatin were associated with an increased risk of uterine haemorrhage, simvastatin was associated with an increased risk of menstruation irregular, and pravastatin was associated with increased risk of menorrhagia and dysfunctional uterine bleeding. Although these uterine bleeding AEs of statins have not been previously reported, the bleeding risk at other sites associated with statins has already been identified. For example, several epidemiologic studies indicated that statin use was associated with increased risk of intracerebral hemorrhage and gastrointestinal hemorrhage^33–35^. The underlying mechanisms by which statins increase bleeding risk may include the following points: first, lower serum cholesterol levels induced by statins may increase risk of rupture and hemorrhage by reducing vessel resistance to tension^36^; second, statins could inhibit the prenylation of small GTP-binding proteins (GTPases), such as CDC42 and Rac1, resulting in alteration of vascular permeability^37^; third, statins also have antithrombotic effects, decrease platelet activity, inhibit platelet aggregation, have anticoagulant activity, affect blood viscosity and RBC deformability, and ‘improve’ von Willebrand factor activity^33^.

Several epidemiologic studies have investigated the effects of statins on sexual function, but generated conflicting results: some studies indicated that statin use was associated with increased risk of sexual function disorders^38,39^, while others found no effects on sexual function^40^. Our study found that statins as a class were associated with increased risk of dyspareunia, sexual dysfunction, disturbance in sexual arousal and orgasm abnormal, atorvastatin was associated with increased risk of dyspareunia, sexual dysfunction and anorgasmia, and simvastatin was associated with increased risk of libido increased, sexual dysfunction and disturbance in sexual arousal. The mechanism for increased risk of sexual function disorders could be partly attributable to the effects of statins on gonadal steroid hormones. Statins may reduce gonadal steroid hormone biosynthesis through hepatic inhibition of cholesterol synthesis, which is the precursors of androstenedione and estradiol^40^. Reductions of gonadal steroid hormones in females are found to be associated with low sexual desire, arousal, and responsiveness^41^.

Our study has several strengths. First, FAERS contains all AE reports submitted to the FDA, and the sample size is sufficient enough to identify rare adverse reactions which could hardly be found in conventional epidemiologic studies^42^. Second, in our study, we detected signals for 383 PTs, which could reflect full AE profiles of statins on the functions of ovary and uterus. Third, as the risks of some ovary and uterus related AEs may vary considerably by age, we also performed subgroup analysis to identify age-specific AEs associated with statins. Thus, our study provided novel and comprehensive insight into the safety of statins on the functions of ovary and uterus.

Despite these strengths, some limitations inherent to the FAERS database should be taken into account when interpreting our results. First of all, the FAERS database has missing data. Some eligible AE reports could not be included in our disproportionality analysis due to the lack of data on gender or age. Moreover, dosage information could not be analyzed in our study, as it was not recorded in most AE reports. Secondly, the results of disproportionality analyses could only demonstrate associations and not causations. Thus, further epidemiologic studies are still needed to verify the causations between statin use and occurrence of AEs.

In conclusion, our analysis of the FAERS database suggests that statin use may be associated with a series of ovary and uterus related AEs. These AEs are involved in ovarian cysts and neoplasms, uterine neoplasms, cervix neoplasms, uterine disorders (excl neoplasms), cervix disorders (excl neoplasms), endocrine disorders of gonadal function, menstrual cycle and uterine bleeding disorders, menopause related conditions, and sexual function disorders. Moreover, there are variabilities in the types and signal strengths of ovary and uterus related AEs across individual statins. Although the mechanisms for most AEs remains unknown, the potential risks of ovary and uterus related AEs associated with statin use are of great importance and should be closely monitored in future clinical practice. Further studies are needed to both confirm our findings and explore the underlying mechanisms.

## Methods

### Data source

FAERS is a spontaneous AEs database designed to support the FDA’s post-marketing safety surveillance system for drug and biologic products approved in the United States. FAERS data are publicly available, and contain patient demographic and administrative information, drug information, AEs, patient outcome, and report sources^42^. In the FAERS database, each report may have one or more AEs. We used the open tool OpenVigil 2.1 (https://www.is.informatik.uni-kiel.de/pvt/OpenVigilMedDRA17/search/) to query the FAERS database. OpenVigil 2.1 is a pharmacovigilance data extraction, cleaning, mining and analysis tool of the FAERS database^43,44^*.* Moreover, it now includes FAERS data from Q4/2003-Q3/2019.

### Query construction

OpenVigil 2.1 has mapped arbitrary drug names (generic names, brand names, abbreviations, and so on) to unique drugnames by using Drugbank and Drugs@FDA. Thus, we firstly identified the drugnames of each statin (atorvastatin, simvastatin, rosuvastatin, pravastatin, lovastatin, fluvastatin and pitavastatin) in the “Browse Window” of OpenVigil 2.1. Then we searched the drugnames of both statins as a class and individual statins in the “OpenVigil Search Window”, respectively. Moreover, in the “Advanced Search Box”, the gender of patient was restricted to female only.

### Definition of ovary and uterus related AEs

In the FAERS database, AEs are coded using Preferred Terms (PTs) in the Medical Dictionary for Regulatory Activities (MedDRA) terminology. In our study, ovary and uterus related AEs were defined by 383 PTs (see Supplementary Table S1 online). Moreover, according to the structural hierarchy of MedDRA terminology, these 383 PTs could be classified into ten aspects: ovarian cysts and neoplasms, uterine neoplasms, cervix neoplasms, ovarian disorders (excl cysts and neoplasms), uterine disorders (excl neoplasms), cervix disorders (excl neoplasms), endocrine disorders of gonadal function, menstrual cycle and uterine bleeding disorders, menopause related conditions, and sexual function disorders^45^.

### Statistical analysis

Disproportionality analyses were done by using OpenVigil 2.1. In the “Data Presentation And Statistics Box” of OpenVigil 2.1, proportional reporting ratio (PRR) was calculated to assess the association between ovary and uterus related AEs and statins. A higher PRR suggests a stronger association, and PRR = 2 indicates that the AE is two times more frequent in users of the drug than in the background population. According to the criteria of Evans et al.^46^, a positive signal of disproportionality was defined as PRR at least two, chi-squared of at least four, and three or more cases. As ovary and uterus related AEs were defined by 383 PTs, we firstly performed 383 disproportionality analyses to assess the associations between these 383 PTs and statins as a class. Then we performed 2,681 disproportionality analyses to assess the associations between these 383 PTs and each statin (atorvastatin, simvastatin, rosuvastatin, pravastatin, lovastatin, fluvastatin and pitavastatin). Moreover, as the risks of ovary and uterus related AEs may be affected by age, we also conducted subgroup analysis stratified by age groups (0–18 years, 19–39 years, 40–59 years, and ≥ 60 years), and in this step 12,256 disproportionality analyses were performed. The age groups were categorized based on different physical phases of the female^47,48^. A total of 15,320 disproportionality analyses were performed in our study, and the details are shown in Supplementary Table S2 online.

## Supplementary information

Supplementary Information.

## Data Availability

The datasets generated during the current study are available from the corresponding authors on reasonable request.
