# Supplementary material for: Ovary and uterus related adverse events associated with statin use: an analysis of the FDA Adverse Event Reporting System
Source: Sci Rep. 2020 Jul 20;10:11955. doi: 10.1038/s41598-020-68906-2 (PMC7371681; doi:10.1038/s41598-020-68906-2)
Supplement: Supplementary file 1 — Supplementary Information. [file 41598_2020_68906_MOESM1_ESM.pdf]

Ovary and uterus related adverse events associated with statin use: an analysis of the FDA Adverse Event Reporting System

Xue-feng Jiao[1, 2, 3, 4, +], Hai-long Li[1, 2, 3, +], Xue-yan Jiao[5], Yuan-chao Guo[1, 2, 3], Chuan Zhang[1, 2, 3], Chun-song Yang[1, 2, 3], Li-nan Zeng[1, 2, 3], Zhen-yan Bo[1, 2, 3], Zhe Chen[1, 2, 3], Hai-bo Song[6*], Ling-li Zhang[1, 2, 3*]

[1] Department of Pharmacy, West China Second University Hospital, Sichuan University, Chengdu 610000, Sichuan, China.

[2] Evidence-Based Pharmacy Center, West China Second University Hospital, Sichuan University, Chengdu 610000, Sichuan, China.

[3] Key Laboratory of Birth Defects and Related Diseases of Women and Children (Sichuan University), Ministry of Education, Sichuan, China.

[4] West China School of Medicine, Sichuan University, Chengdu 610000, Sichuan, China.

[5] Xinxiang Medical University, Xinxiang 453000, Henan, China.

[6] Center for Drug Reevaluation, National Medical Products Administration, Beijing 100000, China.

*e-mail: songhaibo@cdr-adr.org.cn; zhanglingli@scu.edu.cn

[+]These authors contributed equally: Xue-feng Jiao and Hai-long Li.

Page 2-4 **Supplementary Table S1.** Definition of ovary and uterus related adverse events.
Page 5 **Supplementary Table S2.** Number of disproportionality analyses.

Supplementary Table S1. Definition of ovary and uterus related adverse events.

Classification	Preferred Terms (PTs)
Ovarian cysts and neoplasms (n=76)	Benign ovarian tumour, Borderline mucinous tumour of ovary, Borderline ovarian tumour, Borderline serous tumour of ovary, Brenner tumour, Cystadenocarcinoma ovary, Haemorrhagic ovarian cyst, Hyperreactio luteinalis, Leukaemic infiltration ovary, Malignant ovarian cyst, Meigs' syndrome, Metastases to ovary, Mucinous cystadenocarcinoma ovary, Ovarian adenoma, Ovarian cancer, Ovarian cancer metastatic, Ovarian cancer recurrent, Ovarian cancer stage I, Ovarian cancer stage II, Ovarian cancer stage III, Ovarian cancer stage IV, Ovarian clear cell carcinoma, Ovarian cyst, Ovarian cyst ruptured, Ovarian cyst torsion, Ovarian dysgerminoma stage I, Ovarian dysgerminoma stage II, Ovarian dysgerminoma stage III, Ovarian dysgerminoma stage IV, Ovarian epithelial cancer, Ovarian epithelial cancer metastatic, Ovarian epithelial cancer recurrent, Ovarian epithelial cancer stage I, Ovarian epithelial cancer stage II, Ovarian epithelial cancer stage III, Ovarian epithelial cancer stage IV, Ovarian fibroma, Ovarian germ cell cancer stage I, Ovarian germ cell cancer stage II, Ovarian germ cell cancer stage III, Ovarian germ cell cancer stage IV, Ovarian germ cell choriocarcinoma, Ovarian germ cell choriocarcinoma stage I, Ovarian germ cell choriocarcinoma stage II, Ovarian germ cell choriocarcinoma stage III, Ovarian germ cell choriocarcinoma stage IV, Ovarian germ cell embryonal carcinoma stage I, Ovarian germ cell embryonal carcinoma stage II, Ovarian germ cell embryonal carcinoma stage III, Ovarian germ cell embryonal carcinoma stage IV, Ovarian germ cell endodermal sinus tumour, Ovarian germ cell endodermal sinus tumour stage I, Ovarian germ cell endodermal sinus tumour stage II, Ovarian germ cell endodermal sinus tumour stage III, Ovarian germ cell endodermal sinus tumour stage IV, Ovarian germ cell polyembryoma, Ovarian germ cell polyembryoma stage I, Ovarian germ cell polyembryoma stage II, Ovarian germ cell polyembryoma stage III, Ovarian germ cell polyembryoma stage IV, Ovarian germ cell teratoma, Ovarian germ cell teratoma benign, Ovarian germ cell teratoma stage I, Ovarian germ cell teratoma stage II, Ovarian germ cell teratoma stage III, Ovarian germ cell teratoma stage IV, Ovarian germ cell tumour, Ovarian germ cell tumour mixed, Ovarian granulosa cell tumour, Ovarian granulosa-theca cell tumour, Ovarian low malignant potential tumour, Ovarian neoplasm, Ovarian Sertoli-Leydig cell tumour, Ovarian theca cell tumour, Parovarian cyst, Serous cystadenocarcinoma ovary
Uterine neoplasms (n=36)	Benign hydatidiform mole, Benign uterine neoplasm, Clear cell endometrial carcinoma, Endometrial adenocarcinoma, Endometrial adenoma, Endometrial cancer, Endometrial cancer metastatic, Endometrial cancer recurrent, Endometrial cancer stage 0, Endometrial cancer stage I, Endometrial cancer stage II, Endometrial cancer stage III, Endometrial cancer stage IV, Endometrial neoplasm, Endometrial sarcoma, Endometrial sarcoma metastatic, Endometrial sarcoma recurrent, Endometrial stromal sarcoma, Intravenous leiomyomatosis, Malignant hydatidiform mole, Metastases to uterus, Metastatic uterine cancer, Mucinous endometrial carcinoma, Multiple cutaneous and uterine leiomyomatosis, Papillary serous endometrial carcinoma, Placental chorioangioma, Sarcoma uterus, Squamous endometrial carcinoma, Uterine cancer, Uterine carcinoma in situ, Uterine cyst, Uterine leiomyoma, Uterine leiomyosarcoma, Uterine myoma expulsion, Uterine neoplasm, Uterine polyp

Cervix neoplasms (n=18)	Adenocarcinoma of the cervix, Adenosquamous carcinoma of the cervix, Benign neoplasm of cervix uteri, Cervical cyst, Cervical polyp, Cervix adenomatous polyp, Cervix cancer metastatic, Cervix carcinoma, Cervix carcinoma recurrent, Cervix carcinoma stage 0, Cervix carcinoma stage I, Cervix carcinoma stage II, Cervix carcinoma stage III, Cervix carcinoma stage IV, Cervix neoplasm, Clear cell carcinoma of cervix, Small cell carcinoma of the cervix, Squamous cell carcinoma of the cervix
Ovarian disorders (excl cysts and neoplasms) (n=40)	Adnexal torsion, Delayed follicular ripening, Hyperthecosis, Hypogonadism female, Ovarian adhesion, Ovarian atrophy, Ovarian calcification, Ovarian disorder, Ovarian dysgerminoma stage unspecified, Ovarian embryonal carcinoma, Ovarian endometrioid carcinoma, Ovarian enlargement, Ovarian failure, Ovarian failure postoperative, Ovarian fibrosis, Ovarian germ cell cancer, Ovarian haematoma, Ovarian haemorrhage, Ovarian hyperfunction, Ovarian hyperstimulation syndrome, Ovarian mass, Ovarian necrosis, Ovarian oedema, Ovarian prolapse, Ovarian remnant syndrome, Ovarian rupture, Ovarian stromal cancer, Ovarian stromal hyperplasia, Ovarian vein thrombosis, Ovulation delayed, Ovulation disorder, Ovulation pain, Premature follicular ripening, Premature ovulation, Residual ovary syndrome, Superovulation, Varicocele ovarian, Varicocele utero-ovarian, Biopsy ovary abnormal, Ultrasound ovary abnormal
Uterine disorders (excl neoplasms) (n=54)	Adenomyosis, Asherman's syndrome, Decidual cast, Endometrial atrophy, Endometrial disorder, Endometrial dysplasia, Endometrial hyperplasia, Endometrial hypertrophy, Endometrial hypoplasia, Endometrial metaplasia, Endometrial thickening, Endometriosis, Uterine adhesions, Uterine atrophy, Uterine dehiscence, Uterine disorder, Uterine enlargement, Uterine fibrosis, Uterine fistula, Uterine haematoma, Uterine injury, Uterine ischaemia, Uterine mass, Uterine necrosis, Uterine pain, Uterine perforation, Uterine peristalsis absent, Uterine rupture, Uterine scar, Uterine scar diverticulum, Uterine stenosis, Uterine tenderness, Endometrium arrested stage, Haematocoele female, Hydrocele female, Uterine inversion, Uterine malposition, Uterine obstruction, Incoordinate uterine action, Postpartum uterine subinvolution, Uterine atony, Uterine contractions abnormal, Uterine hyperstimulation, Uterine hypertonus, Uterine hypotonus, Uterine spasm, Hysterocele, Uterine prolapse, Uterovaginal prolapse, Biopsy endometrium abnormal, Biopsy uterus abnormal, Hysterosalpingogram abnormal, Hysteroscopy abnormal, Ultrasound uterus abnormal
Cervix disorders (excl neoplasms) (n=25)	Cervical bulla, Cervical discharge, Cervical dysplasia, Cervical friability, Cervical incompetence, Cervical leukoplakia, Cervix disorder, Cervix enlargement, Cervix erythema, Cervix haematoma uterine, Cervix haemorrhage uterine, Cervix oedema, Ectropion of cervix, Shortened cervix, Uterine cervical erosion, Uterine cervical exfoliation, Uterine cervical laceration, Uterine cervical pain, Uterine cervical squamous metaplasia, Uterine cervix atrophy, Uterine cervix hypoplasia, Uterine cervix stenosis, Uterine cervix ulcer, Smear cervix abnormal, Biopsy cervix abnormal
Endocrine disorders of gonadal function (n=75)	Adiposogenital dystrophy, Antigonadotrophins present, Gonadotrophin deficiency, Gonadotrophin releasing hormone deficiency, Olfacto genital dysplasia, Secondary hypogonadism, Androgenetic alopecia, Antigonadotrophins present, Congenital androgen deficiency, Gonadotrophin deficiency, Hyperandrogenism, Hypergonadism, Hyperprogesteronism, Hypogonadism, Hypoprogesteronism, Oestrogen deficiency, Oestrogenic effect, Primary

	hypogonadism, Secondary sexual characteristics absence, Delayed puberty, Incomplete precocious puberty, Precocious puberty, Pseudoprecocious puberty, Pubertal failure, Virilism, Virilism foetal, 17 ketosteroids urine abnormal, 17 ketosteroids urine decreased, 17 ketosteroids urine increased, Androgens abnormal, Androgens decreased, Androgens increased, Anti-Muellerian hormone level decreased, Anti-Muellerian hormone level increased, Blood androstenedione decreased, Blood androstenedione increased, Blood oestrogen abnormal, Blood oestrogen decreased, Blood oestrogen increased, Blood testosterone abnormal, Blood testosterone decreased, Blood testosterone free abnormal, Blood testosterone free decreased, Blood testosterone free increased, Blood testosterone increased, Dihydrotestosterone decreased, Dihydrotestosterone increased, Female sex hormone level abnormal, Free androgen index decreased, Oestradiol abnormal, Oestradiol decreased, Oestradiol increased, Oestriol abnormal, Oestriol decreased, Oestriol increased, Oestrogens total urine abnormal, Oestrogens total urine decreased, Oestrogens total urine increased, Oestrone decreased, Oestrone increased, Progesterone abnormal, Progesterone decreased, Progesterone increased, Saliva testosterone abnormal, Saliva testosterone decreased, Saliva testosterone increased, Salivary oestriol increased, Sex hormone binding globulin decreased, Sex hormone binding globulin increased, Adrenal androgen deficiency, Adrenal androgen excess, Adrenogenital syndrome, Albright's disease, Follicle-stimulating hormone deficiency, Luteinising hormone deficiency, Polycystic ovaries
Menstrual cycle and uterine bleeding disorders (n=28)	Uterine haemorrhage, Abnormal withdrawal bleeding, Anovulatory cycle, Bleeding anovulatory, Delayed menarche, Dysfunctional uterine bleeding, Dysmenorrhoea, Menstrual discomfort, Menstrual disorder, Menstruation irregular, Metrorrhagia, Premature menarche, Premenstrual cramps, Premenstrual dysphoric disorder, Premenstrual headache, Premenstrual pain, Premenstrual syndrome, Retrograde menstruation, Withdrawal bleed, Amenorrhoea, Hypomenorrhoea, Menstruation delayed, Oligomenorrhoea, Pituitary amenorrhoea, Menometrorrhagia, Menorrhagia, Polymenorrhagia, Polymenorrhoea
Menopause related conditions (n=9)	Artificial menopause, Hot flush, Menopausal depression, Menopausal disorder, Menopausal symptoms, Menopause delayed, Premature menopause, Radiation menopause, Postmenopausal haemorrhage
Sexual function disorders (n=22)	Fertility increased, Dyspareunia, Female orgasmic disorder, Female sexual arousal disorder, Female sexual dysfunction, Infertility, Infertility female, Libido decreased, Libido disorder, Libido increased, Orgasm abnormal, Orgasmic sensation decreased, Sexual dysfunction, Anorgasmia, Disturbance in sexual arousal, Inadequate lubrication, Sexual inhibition, Hypersexuality, Loss of libido, Antral follicle count high, Antral follicle count low, Infertility tests abnormal

Supplementary Table S2. Number of disproportionality analyses.

Number of disproportionality analyses (n) / Age groups (years)	Statins as a class	Atorvastatin	Simvastatin	Rosuvastatin	Pravastatin	Lovastatin	Fluvastatin	Pitavastatin
Overall	383	383	383	383	383	383	383	383
0-18	383	383	383	383	383	383	383	383
19-39	383	383	383	383	383	383	383	383
40-59	383	383	383	383	383	383	383	383
≥60	383	383	383	383	383	383	383	383

Red font: Number of disproportionality analyses for statins as a class.

Green font: Numbers of disproportionality analyses for each statin.

Blue font: Numbers of disproportionality analyses for subgroup analyses stratified by age groups.
